# Rapid Quaternary subsidence in the northwestern German North Sea

**DOI:** 10.1038/s41598-018-29638-6

**Published:** 2018-08-01

**Authors:** Jashar Arfai, Dieter Franke, Rüdiger Lutz, Lutz Reinhardt, Jonas Kley, Christoph Gaedicke

**Affiliations:** 1 Federal Institute for Geosciences and Natural Resources (BGR), Stilleweg 2, 30655 Hannover, Germany; 2 Georg August University Göttingen, Goldschmidtstraße 3, 37077 Gottingen, Germany

## Abstract

3D and 2D seismic data reveal the base-reflection of the Quaternary in the northwestern German North Sea locally at depths of more than 1000 m. This indicates extremely fast subsidence, with a rate of up to 480 m/Ma during the Quaternary, resulting in a NNW-SSE oriented sedimentary depocentre. Distinct iceberg scour marks, identified in 3D seismic data are used to calibrate quantitative subsidence analysis and to document shallow marine conditions during the Quaternary interglacials. Previously, a number of mechanisms have been proposed to explain the Quaternary subsidence. Here we show that compaction and load-induced subsidence alone explain about 75% of the observed Quaternary subsidence. However, a certain portion of the subsidence needs additional processes to be invoked. The extensive seismic dataset interpreted here makes it possible to exclude a phase of renewed tectonic activity as the origin of the subsidence anomaly. From the orientation and extent of the depocentre, lithosphere buckling and subsidence due to salt movement are considered unlikely. Possibly a post-glacial collapse after the retreat of glaciers in the North Sea Basin, local lower crustal flow, or dynamic topography or a combination of these processes contributed to the residual subsidence.

## Introduction

**Figure 1:**
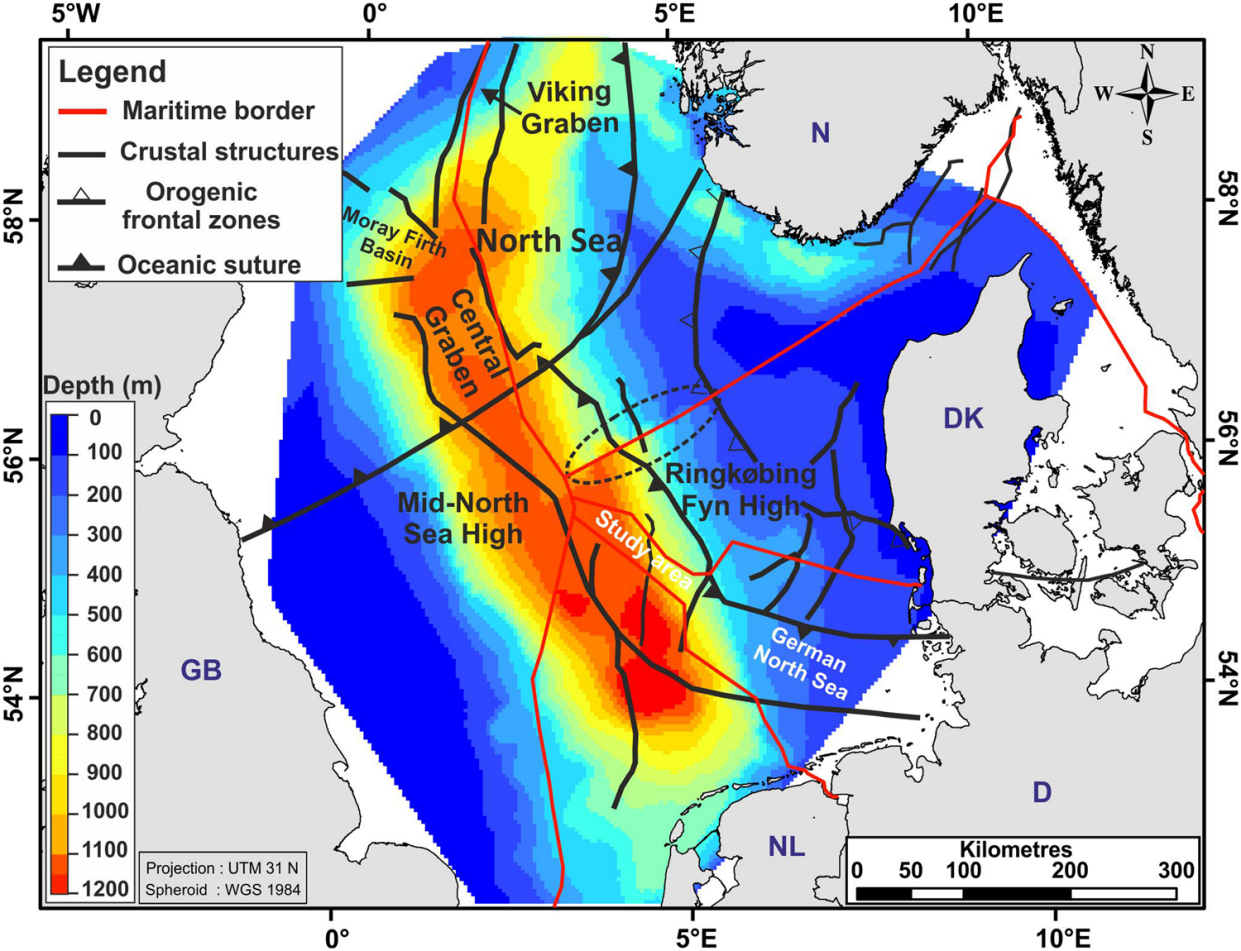
Colour coded depth map of the base Quaternary surface in the North Sea. The study area in the northwestern German offshore area covers an area of approximately 4000 km². Data and crustal structures are based on the published maps from the Southern Permian Basin Atlas^33^ (by permission), study on the northern North Sea^45^ (by permission) area and our study (Fig. 2). The dotted ellipsoid indicates a mismatch at the border between the Danish and Norwegian North Sea. Land polygons ©OpenStreetMap contributors^65^ (available under the Open Database License; see www.openstreetmap.org/copyright).

**Figure 2:**
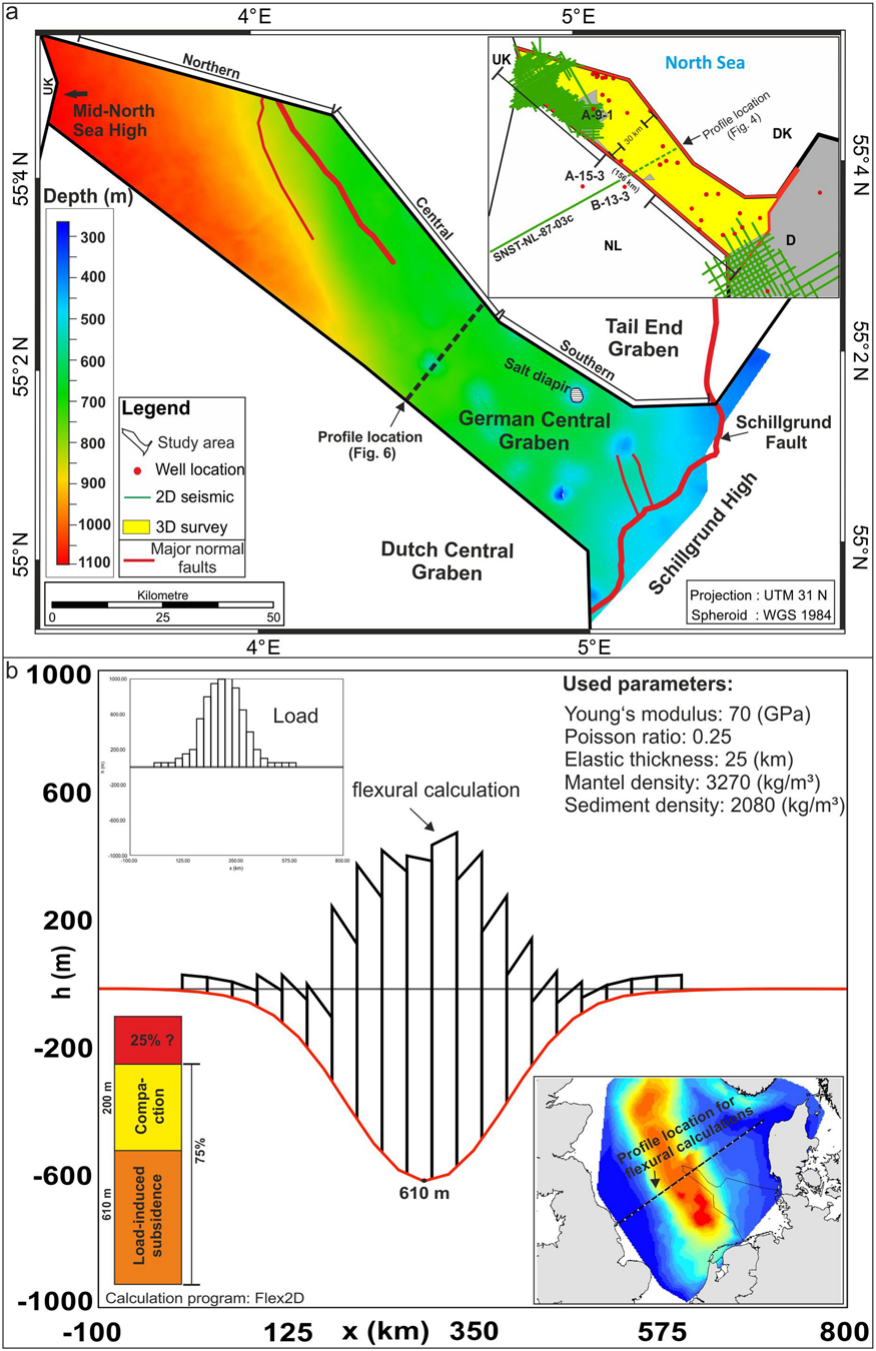
Colour coded depth map of the base Quaternary surface offshore northwestern Germany. The main Mesozoic structural elements are based on a detailed previous study^54^. The inset shows the data source, 3D surveys (yellow) and locally 2D seismic datasets (green lines) and 29 exploration wells (red dots). The location of the SNST-Nl-87-03c 2D line^40^ and the wells A-15-3 and B-13-3 in the Dutch North Sea area are indicated, (**a**) Flexural calculation on a SW-NE profile across the depocentre (see inset, lower right corner), (**b**) by taking flexure into account, the proportion of observed subsidence not caused by loading is greater than 20%.

The widely accepted McKenzie model^1^ predicts strong subsidence during the extension phase of continental rifts. During the subsequent post-rift phase, subsidence theoretically^1^ declines exponentially. The Mesozoic/Early Cenozoic subsidence pattern of the North Sea is interpreted as a result of thermally induced subsidence following Late Triassic to Early Cretaceous rifting events accompanied by sediment loading and isostatic adjustments^2–7^. However, during the Quaternary (2.6 Ma), the Central Graben area of the North Sea rift experienced an unusually high degree of subsidence (i.e., anomalous subsidence), at about 140 Ma after the final rifting phase^2,3^. During these 2.6 Ma, a thick package of more than 1000 m of sediments accumulated in the North Sea Basin, including the northwestern German North Sea (Figs 1 and 2). Various potential mechanisms have been proposed as causes of the anomalously high rate of Quaternary subsidence^2,3,8–10^. These include reactivation of faulting in association with a renewed rifting phase^2,3^, lithosphere buckling due to intraplate stresses^8–11^, metamorphic processes at the crust-mantel transition^12^, and the influence of a mantle thermal anomaly^13,14^. More generally, other modes of mantle convection are also suggested as causing dynamic topography, i.e. topography deviating from values corresponding to isostatic equilibrium^15^.

Alongside the distinct offshore subsidence, onshore areas around the North Sea are subject to large-scale uplift^16–21^. In eastern Sweden, an isostatic uplift of about 310 m since the deglaciation of the last glacial maximum has been derived from Holocene shore displacement investigations by radiocarbon-dating^22^. It is suggested that the topography and landscape in northwestern England have almost entirely formed since the Middle Pliocene, accompanying ~800 m or more of uplift, while further to the south, total uplift since the Middle Pliocene has been estimated to be in the range of 150–200 m^23,24^. However, it is questionable if the uplift occurred during the Quaternary or actually before the Pleistocene^25,26^. Nevertheless, the widespread Neogene uplift around the North Sea (and beyond) has been suggested to be linked to local offshore subsidence via enhanced sediment supply^9,16^ or by lower crustal flow^10,17,23,27^.

In this study, we calibrate the Quaternary succession in time and (palaeo-) depth by using iceberg scour marks, indicating shallow marine conditions during the interglacial periods. The Quaternary depocentre is regionally interpreted to be in the northwestern German North Sea, supplemented by published depth maps around northwestern offshore Germany. We calculated the well-constrained processes such as compaction and load-induced subsidence, and conclude that only about 25% of the observed subsidence requires additional explanation. This is discussed in the light of earlier suggested mechanisms for the high Quaternary subsidence.

## Geological setting of the North Sea Basin

The Cenozoic North Sea Basin developed as an intracratonic sag basin and is centred above Mesozoic rift structures, including the Central Graben^28–30^ (Figs 1 and 2). The Cenozoic depocentre, which is aligned along a NW-SE axis, preserves more than 3 km of Cenozoic, mainly siliciclastic sediments^9,31,32^.

The Quaternary sedimentary succession reaches a maximum thickness of more than 1000 m along a NNW-SSE trending basin axis in the central North Sea^33^ (Fig. 1). Because most Quaternary sediments were deposited in a shallow-water environment^2,34^, the southern part of the North Sea Basin must have locally experienced an extremely high rate of subsidence exceeding average Cenozoic rates by a factor of 10, as revealed by Cenozoic thickness maps^2,31,32^.

The Quaternary North Sea Basin is characterized by three extensive glaciations, which covered large parts of the North Sea and adjacent land areas during the Pleistocene^35–38^.

**Figure 3:**
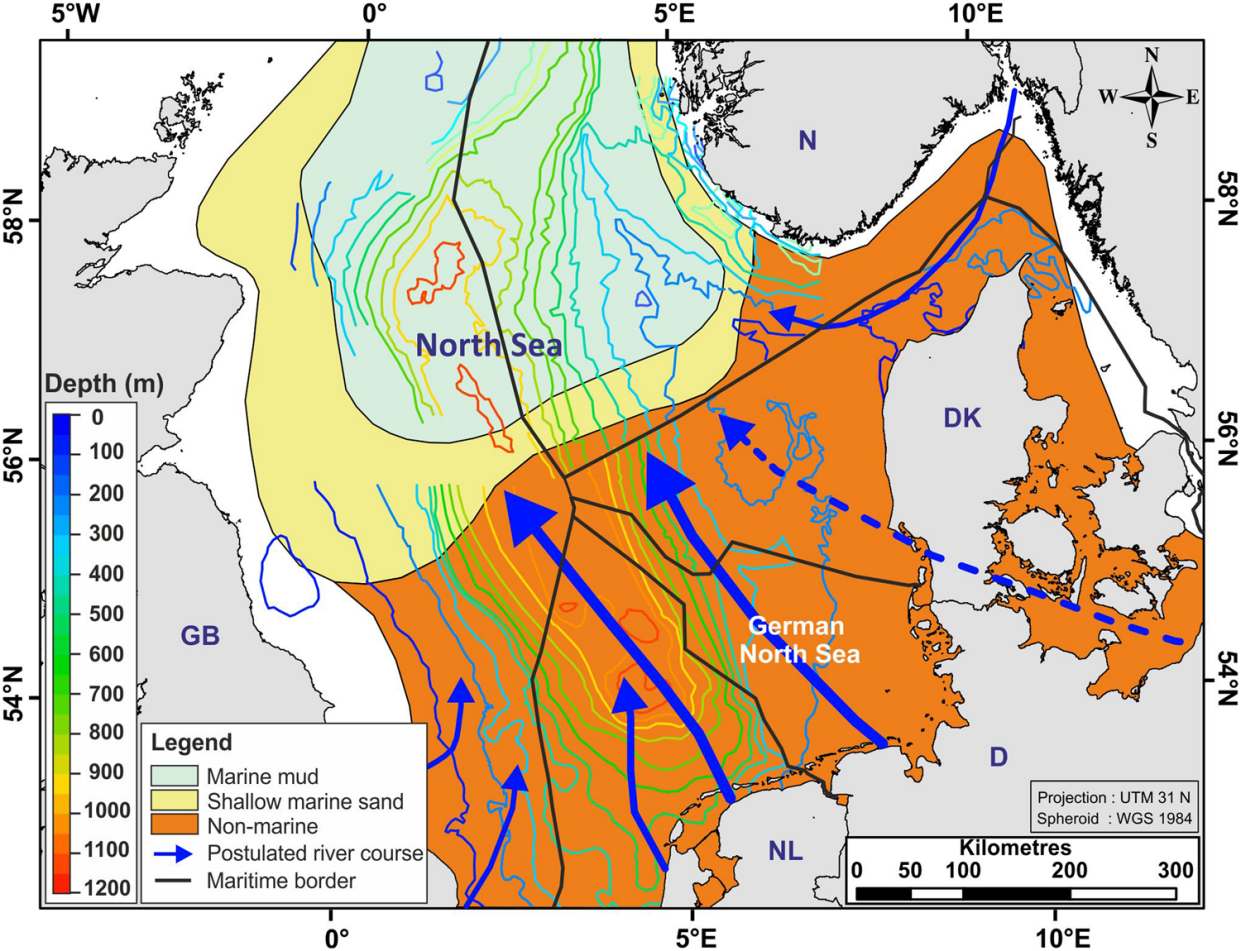
Quaternary palaeogeography modified from the Southern Permian Basin Atlas^33^ (by permission). Blue arrows indicate postulated river courses and sediment supply directions during the Quaternary^33^. Contours show the thickness of the Quaternary strata (Fig. 1). Land polygons ©OpenStreetMap contributors^65^ (available under the Open Database License; see www.openstreetmap.org/copyright).

The change between glacial and interglacial periods during the Quaternary resulted in strong sea-level fluctuations, with amplitudes between 100–150 m, and coastline shifts with a vertical range of up to 120 m around the North Sea^18,34^. Consequently, non-marine conditions were repeatedly established in portions of the southern North Sea during the Pleistocene (Fig. 3), as evidenced by subglacial tunnel valleys^39^ within the uppermost Quaternary section. Repeated regressive phases coupled with climatic deterioration, led to increased sediment supply mainly from the south, via the rivers Saale, Elbe, Weser, and Rhine, which provided the bulk of the sediments that accumulated in the southern North Sea area during the Quaternary^10,33^ (Fig. 3).

## Interpretation and Results

### Glacial features

**Figure 4:**
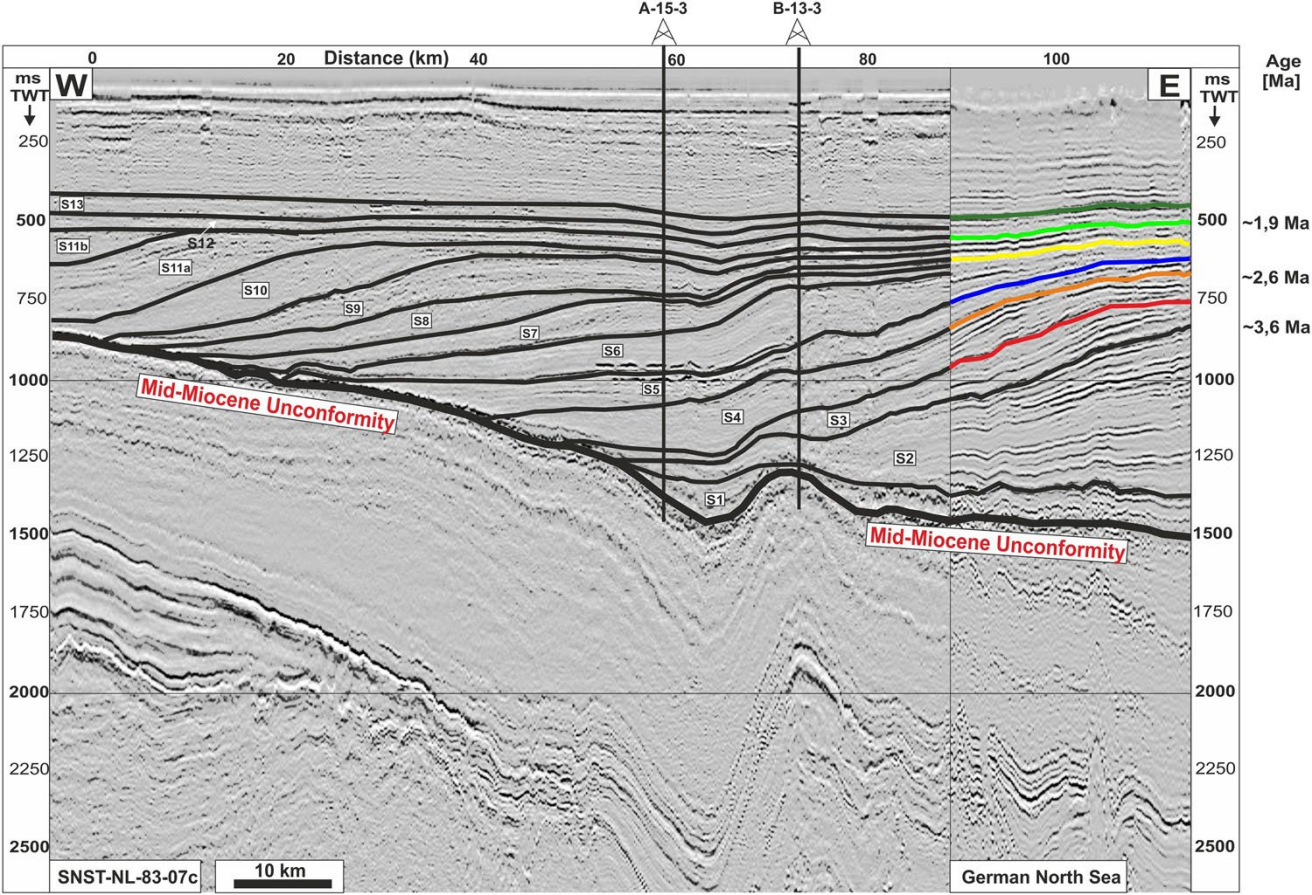
W-E oriented composite 2D seismic profile (see Fig. 2 for location), running from the Dutch^40^ into the German North Sea. The stratigraphy is tied to a detailed age model based on wells A-15-3 and B-13-3^40^ (see Fig. 2 for location). 10 sub-units (S4–S13) above the Mid-Miocene Unconformity (MMU) including inter-Quaternary surfaces are traced through a 3D seismic survey into offshore northwestern Germany. Six interpreted surfaces (coloured lines) are associated with distinct iceberg scour marks. This study is based mainly on two of the horizons (orange and light green line) with ages of 2.6 and 1.9 Ma respectively. The orange line reflects the base Quaternary horizon. Seismic profile: courtesy of Wintershall Holding GmbH.

**Figure 5:**
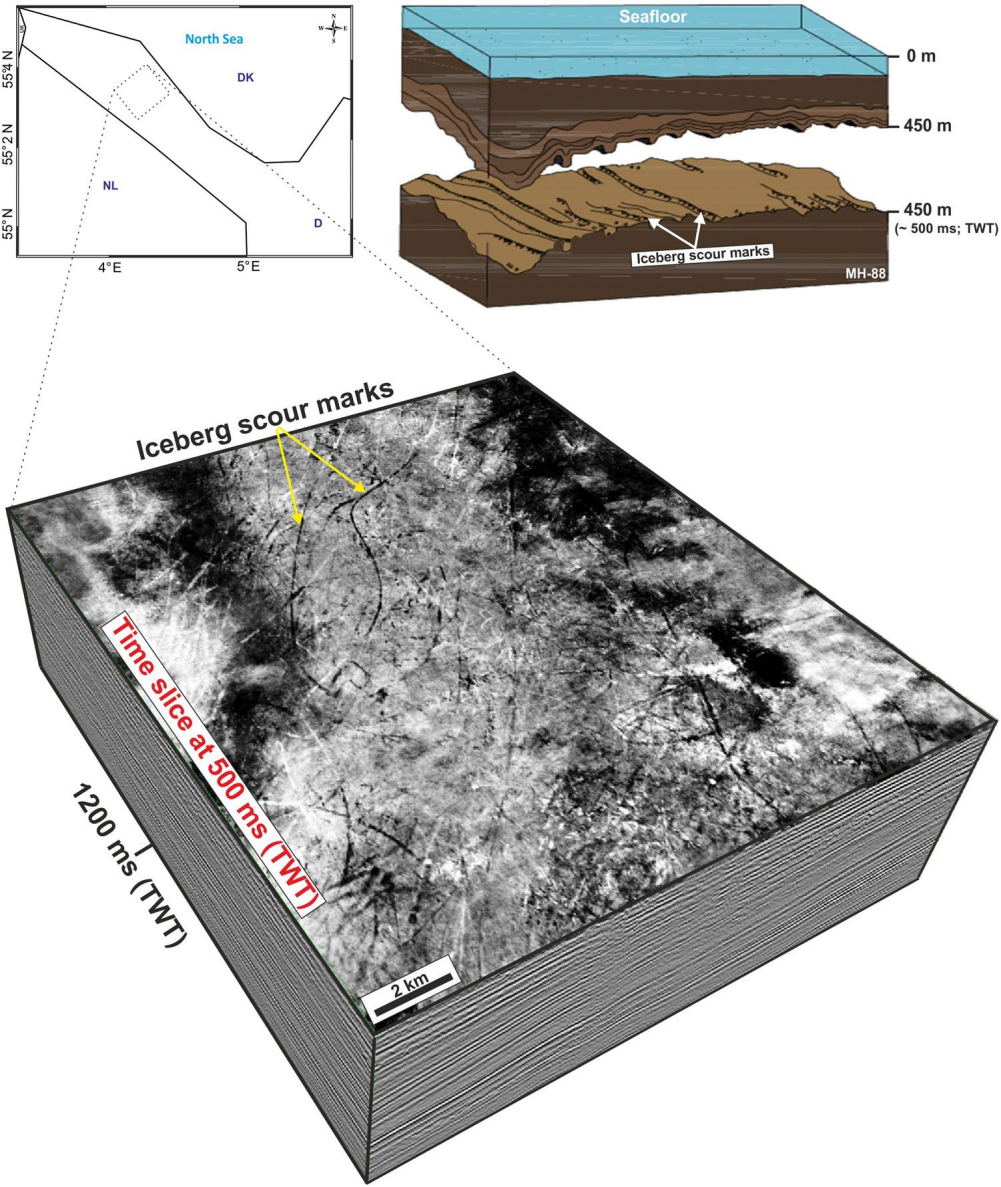
Time slice with distinct curvilinear and cross-cutting lineations interpreted as iceberg scour marks at 500 ms (TWT; ~450 m). The time-depth conversion of interpreted seismic reflectors is based on a generalized linear relationship that reflects depth due to burial and compaction^54^. The modified sketch^42^ (by permission) in the upper right illustrates iceberg scour marks on the former sea floor which is now buried to a depth of about 450 m. These glacial features are used to calibrate the Quaternary subsidence pattern and subsidence rates within the German North Sea.

Eight prominent seismic horizons between 250–1000 ms depth (TWT, approximately 200–1000 m; see chapter Methods and stratigraphic correlation) are interpreted in the study area. All reveal curvilinear and cross-cutting lineations, interpreted as iceberg scour marks **(**Figs 4 and 5**)**. We consider this to be a robust indication that the surfaces formed under shallow marine conditions with water depths not exceeding 100 m^34^. The distinct lineations exhibit lengths between 0.1 km and 23 km, widths between 15–50 m, and 6–12 m deep incisions. Such structures are well-documented from a variety of present-day and formerly glaciated marine environments^40–45^ and are certainly the result of scraped iceberg-keels into soft sediments on the seabed. The iceberg scour marks are found at a present-day depth between 300 to 850 m with the majority located at depths between 500–700 m **(**Fig. 5**)**. They locally cross-cut each other at a wide range of orientations but regionally show a distinct NNW-SSE to N-S orientation (Fig. 5).

A cross-correlation of two of the interpreted horizons with results from a high-resolution 2D-seismic survey, calibrated by well data from the Dutch offshore (see Fig. 2 for location), reveals ages of these horizons of 1.9 and 2.6 Ma. As previously suggested for the Dutch offshore^40^, the iceberg scour marks in the study area can be attributed to Gelasian (2.6–1.8 Ma) and to Pleistocene (1.8–0.01 Ma) times (Fig. 5). These glacial features on the former sea floor are used in the following as chronological markers, and thus as measures for the Quaternary subsidence pattern and subsidence rates within the German North Sea. Furthermore, interpreted glacial features enable interpretations of the palaeobathymetry and to distinguish between marine and shallow marine conditions.

### Base Quaternary

The present-day depth of the base Quaternary succession in the study area ranges between ~110 m in the southeast, and up to 1100 m towards the central North Sea Basin (Fig. 2). The seismic horizon dated at 2.6 Ma in the Dutch offshore sector^40^ defines the base Quaternary in the German North Sea (Figs 2 and 4). Based on the calculated Quaternary thickness map, a subsidence rate of approximately 410 m/Ma is derived at the location of well A-9-1. This is in sharp contrast to a calculated subsidence rate of ~140 m/Ma for the main Neogene depocentre (240 m/Ma for the Pliocene) above the Central Graben area. These values show that subsidence was higher during the Quaternary, but also point to a trend of increasing subsidence rates already during the Neogene, particularly during the Pliocene. We derive a subsidence rate of 80 m/Ma for the Palaeogene succession from calculated thickness maps in the most northwestern part of the study area. Although subsidence already started to accelerate in the Neogene, the Quaternary subsidence is still exceptional with a more than ten-fold increase in subsidence compared to average Cenozoic sedimentation rates. The Quaternary isopach map (Fig. 2) further illustrates an increase in subsidence from the southeastern part of the study area (180–265 m/Ma) towards the central North Sea Basin, reaching a maximum rate of 480 m/Ma. The main Quaternary depocentre within the central North Sea follows a distinct NNW-SSE trend. It is clearly offset from the Neogene depocentre and is shifted towards the northwest. We calculated a subsidence rate of ~550 m/Ma for the interval 2.6 Ma–1.9 Ma and ~300 m/Ma for 1.9 Ma–0 Ma, respectively at the location of well A-9-1. The thickness of the Quaternary to Holocene sedimentary succession increases from the southeastern to the northwestern part of the study area, where it reaches a maximum of 1045 m.

In the following we consider a range of mechanisms to explain the Quaternary subsidence pattern which can be addressed on the basis of our comprehensive thickness dataset.

### Load-induced subsidence during the Quaternary

In order to decipher the origin of the enormous Quaternary subsidence, first the range of subsidence induced by the load of the sedimentary successions was estimated.

#### Airy isostasy

Values for subsidence were calculated, assuming local loading (Airy model) at well locations, using the following equation^46^:

*y* is the amount of subsidence in the absence of water load, S* is the sediment thickness (depends on well location), *ρ*_*s*_ (2080 *kg/m*^3^) and *ρ*_*m*_ (3270 *kg*/*m*^3^*)* are the mean densities of the sediments and mantle respectively^47,48^.

1 $$y={S}^{\ast }\frac{{\rho }_{s}}{{\rho }_{m}}$$

Our calculations, based on 29 exploration wells covering the study area, show that subsidence due to sediment loading during the Quaternary ranges between approximately 250 m in the southern part of the study area to a maximum of 550 m at the location of the A-9-1 well, and increases towards the NW to about 665 m (Fig. 2a).

#### Flexural response of the crust to loading

By taking flexure into account (Fig. 2b), the amount of subsidence due to sediment loading observed in the study area is slightly reduced if compared to the Airy isostasy model^46^. The flexural response of the lithosphere is modelled as an elastic beam using the following equation^49,50^: Where D is the strength of the elastic lithosphere, E is Young’s Modulus (70 GPa), ν is Poisson’s ratio (0.25), and T is the thickness of the elastic lithosphere (25 km^50^).

2 $$D={E}^{\ast }{T}^{3}/({12}^{\ast }(1-{\nu }^{2}))$$

The 2D flexural model is calculated every 30 km along an about 600 km long SW-NE profile (Fig. 2b). Mean densities of the sediments and mantle are *ρ*_*s*_ (*2080* *kg/m³*) and *ρ*_*m*_ (*3270* *kg/m³*), respectively. The maximal subsidence due to sediment loading by flexure is about 610 m, showing a difference of 55 m to the Airy isostasy model^46^ (Fig. 2b).

Towards the basin edge, where the thickness of the Quaternary decreases to about 100 m, subsidence due to loading using the Airy isostasy model is less than the flexural response of the crust. We consider the thickness of the elastic lithosphere of 25 km^50^ to be reasonable. To get a feeling for the uncertainties, we additionally modelled with values of 10 km and 30 km flexural crustal thickness. Using 10 km flexural crustal thickness, the local isostasy and the flexural response of the crust are almost identical. By using a constant elastic thickness value of 30 km, the maximal difference between the Airy isostasy model and the flexural response of the crust increases and amounts to about 80 m within the depocentre.

In conclusion, isostasy and flexural modelling show that a large amount (75%) of the observed overall Quaternary subsidence is explained by load-induced subsidence without invoking additional mechanisms.

### Sediment compaction

**Figure 6:**
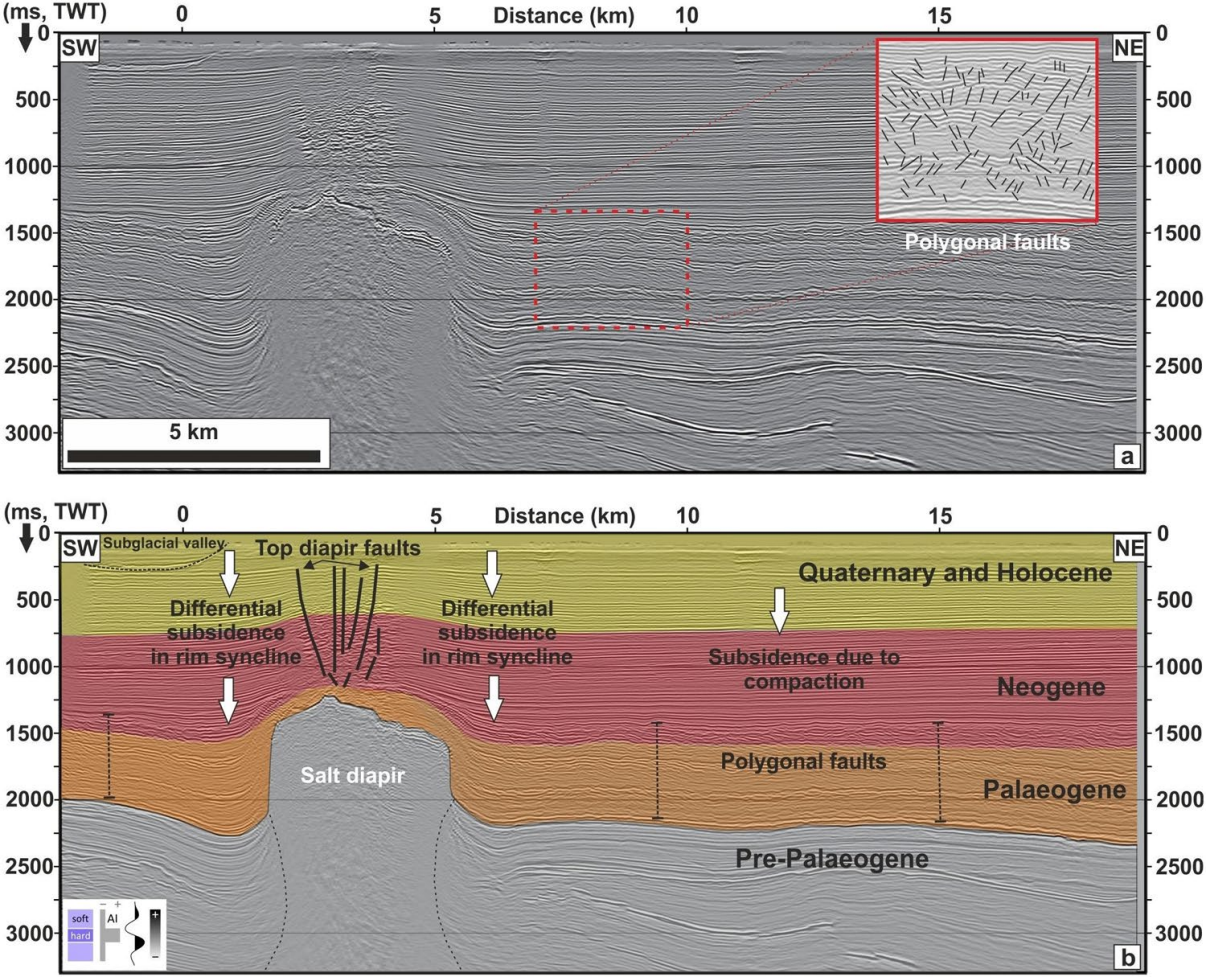
SW-NE oriented seismic profile showing the uppermost Cenozoic sedimentary succession in the study area (see Fig. 2 for location). Salt flow results in deformation of overlying and adjacent sediments through the formation of top diapir faults (crestal collapse faults) and rim synclines. The latter are characterised by differential subsidence around salt diapirs. The compaction of underlying Neogene sediments and particularly Palaeogene strata, characterised by the development of polygonal fault systems (red inset) provide additional accommodation space for sedimentary deposition. Seismic profile: courtesy of Wintershall Holding GmbH.

The compaction of Neogene sediments, and particularly Palaeogene strata underlying the Quaternary succession, is another process that probably contributes to the Quaternary subsidence. Extensive polygonal fault systems^51,52^ in Neogene and Paleogene successions **(**Fig. 6**)** are interpreted here as expressions of compaction, which provides additional regional accommodation space. Based on a 3D basin and petroleum system model^53^, and by considering a lithology of 75% shale (initial porosity, 70%), 15% silt (initial porosity 55%), and 10% sandstone (initial porosity 41%), compaction values range between 150–250 m for the Neogene and Palaeogene strata. This compaction of Palaeogene and Neogene sediments shows an increasing trend from the southern part of the study area towards the northwest, with maximum compaction below the thickest accumulations of Quaternary sediments^53^.

Adding these values to the load-induced subsidence (flexure included) of up to 610 m derived here, results in a combined subsidence of up to 810 m. It is concluded that within the errors, most of the deepening and thus accommodation is controlled and can be explained by load-induced subsidence and compaction.

### Subsidence due to renewed rifting activity during the Quaternary?

The strong Quaternary subsidence phase has been attributed previously to thermal rejuvenation in association with a renewed rifting phase and tectonic activity^2,3^. The foundation of this assumption is primarily the observation that the Quaternary depocentre with a thickness of up to 1200 m partly coincides with older tectonic trends such as the Dutch Central Graben (Fig. 1). However, the same does not consistently apply to the German part of the Central Graben (Figs 1 and 2**)**, where the maximum Quaternary thickness attains 700 m, and the main depocentre is considerably offset (50 km) from Mesozoic structures (Figs 1 and 2). In fact, the main Mesozoic rift structure in the German Central Graben area experienced less Quaternary subsidence compared to the neighbouring areas^54^.

Further southeast and onshore, in northern Central Europe, numerical simulations determined that climate-induced melting of large ice sheets after the Weichselian glaciation was able to trigger fault reactivation and earthquakes around the migrating ice limit^55^. Even today, the stress due to glacial isostatic adjustment can continue to induce seismicity within the once glaciated region^55^.

**Figure 7:**
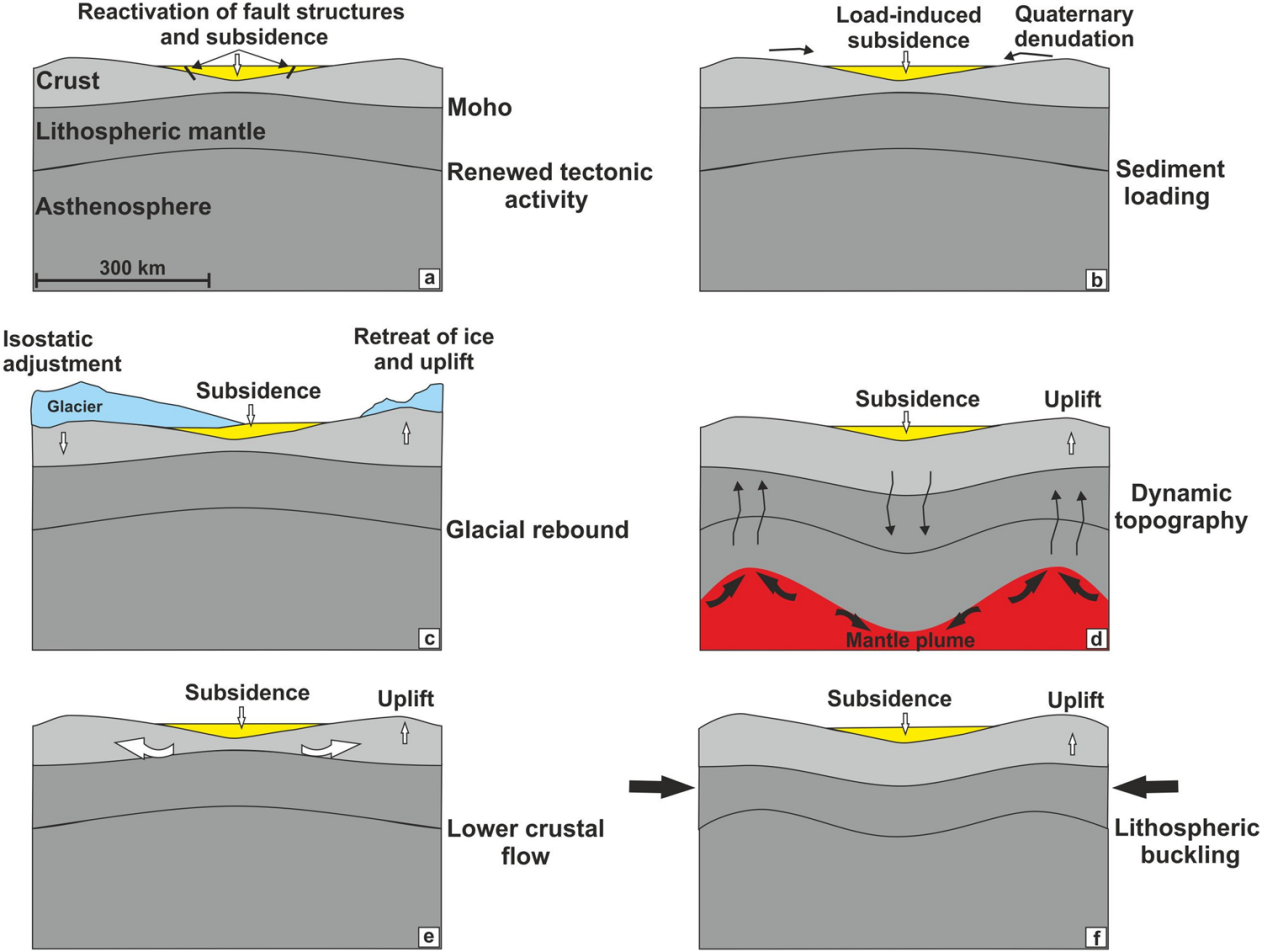
Proposed mechanisms explaining anomalous subsidence in the North Sea: (**a**) renewed tectonic activity, (**b**) load-induced subsidence, (**c**) glacial rebound due to retreat of ice, (**d**) dynamic uplift of lithosphere by upwelling of asthenosphere, (**e**) accelerated subsidence mediated by lower crustal flow and enhanced sediment loading, (**f**) lithospheric buckling due to horizontal stresses.

However, in our extensive offshore seismic data there are no indications of renewed tectonic activity (Fig. 7a) e.g. extensional faulting or the development of pull-apart basins^2,3^. The only exceptions are isolated active salt diapirs and related top diapir fault structures (Fig. 6). Salt-related structures, however, do not coincide spatially with the main Quaternary depocentre but are concentrated in the area of Mesozoic rifting. The Quaternary sedimentary succession is found to be fairly homogenous and is not affected by any significant characteristic tectonically induced fault pattern and/or the reactivation of older Mesozoic tectonic trends. Distinct Mesozoic fault structures, which control the base Upper Cretaceous structural relief, die out upwards within Upper Cretaceous strata. Only a few faults show continuous displacement growth at base Palaeogene, whereas the majority of these faults are associated with salt activity, and a few may be attributed to the abated compressional tectonics at the end of the Late Cretaceous-Early Palaeocene^33,56–59^. Admittedly, our seismic data has a vertical seismic resolution of ~10 m and thus is insufficient to image small-scale faults. However, if there are reactivated faults with offsets of only a few meters which are not resolved in our seismic data, than these faults provide a very minor accommodation space in the North Sea Basin. Overall, however, a renewed rifting phase and fault reactivation in the southern North Sea are considered to be an unlikely origin for the Quaternary subsidence.

### Subsidence due to salt movement

Salt flow results in the deformation of overlying and adjacent sediments through the formation of rim synclines, characterized by distinct angular unconformities. However, such rim synclines accompanied by differential subsidence patterns are exclusively observed locally around salt diapirs (Fig. 6). Thus, diapirism cannot explain the large-scale subsidence pattern in the North Sea. The effect of subsidence induced by salt expulsion or salt flow is also unlikely because the main Quaternary depocentre does not match the area dominated by salt tectonics or by active salt diapirs (Fig. 2). In contrast to the Central Graben area, the main Quaternary depocentre is found in an area with minor salt.

## Discussion

In the previous sections we have shown that most of the subsidence can be explained by compaction and load-induced subsidence. In contrast, a renewed rifting phase, as well as subsidence due to salt mobilization, are unlikely to have contributed to the Quaternary subsidence pattern.

In the following chapter we discuss qualitatively additional processes that may be invoked as an explanation for about 25% of the observed residual subsidence (Fig. 7b).

### Glacial rebound and glacial forebulge collapse

The late Cenozoic uplift of continental areas around the North Atlantic is typically explained by isostatic adjustments following glaciation (Fig. 7c). Unloading due to melting of the Quaternary ice sheets is proposed to result in inflow of mantle in order to re-establish equilibrium, referred to as glacial rebound^17,18,21^.

However, the timing and magnitude of the glacial rebound are under discussion. Two significant Cenozoic episodes of uplift are reported around the North Atlantic, and the second phase occurred during the late Neogene and the Quaternary^16^. It is suggested that Neogene uplift in e.g. the eastern Danish North Sea was caused by isostatic adjustment of the crust as a result of accelerated denudation of up to 1000 m of topography, which was driven by climatic changes and long term eustatic lowstand^16,31,32^. However, there are many observations that indicate that the onset of uplift of landmasses around the North Sea area had already occurred before the first glaciation at 5.1–5.0 Ma^2,16,25,26,31,32^, questioning a causal relationship. The situation is further complicated by the fact that the offshore areas were ice-covered as well but were obviously experiencing subsidence rather than uplift.

The collapse of a peripheral glacial forebulge^60,61^, which developed during the Last Glacial Maximum (LGM) around the Fennoscandian ice sheet may have contributed to the isostatic subsidence. The orientation of this forebulge is reconstructed from models and observational data^60^. It is proposed to have been centered in the North Sea between Norway and Great Britain, extending through northwest Netherlands and northern Germany^60,61^, fitting the observed subsidence pattern in offshore Germany quite well. The main problem we see in invoking such a process is that the collapse of this peripheral forebulge could have created accommodation space for the post-glacial sediments only from 17 ka BP onward and thus may only explain a small portion of the whole Quaternary subsidence pattern. Quantification is difficult because it depends amongst other things on the glacier thickness and extent during the LGM, which are still widely discussed. By assuming an ice sheet with a thickness of about 2800–3700 m^18^ covering the Fennoscandian Shield, the roughly estimated collapse-induced accommodation space would be in a range between 50 m to a maximum of 85 m.

### Subsidence caused by mantle flow

The observation of more or less synchronous onshore uplift and increased subsidence within the North Sea Basin has led to the suggestion of a linkage between both processes^10,16,62^.

Small-scale sub-lithospheric mantle convection is suggested to result in an alternating pattern of rapid Neogene uplift and subsidence across north-west Europe^13,14^ (Fig. 7d). Residual topography, either calculated as deviations from expected water-loaded depths to oceanic basement as a function of plate age or estimated from satellite free-air gravity data, is observed down to wavelengths of about 1300 km^15^. At least some of these positive (uplift) and negative (subsidence) topographic anomalies are proposed to grow and decay on timescales of millions of years^15^. It is believed that they are generated and maintained by circulation of the sub-lithospheric mantle, probably involving rapid flow of low-viscosity material in shallow asthenospheric channels^15^. Nevertheless, at present it appears impossible to either confirm or disprove such a model at the small scale of the North Sea Basin.

### Lower crustal processes

Flow in the lower continental crust is considered another mechanism that potentially isostatically compensates for surface loading of the Earth^10,17,23,27^. Lower crustal flow has e.g. been advocated to explain accelerated phases of basement subsidence in sag basins^27^. Phases of fast erosion onshore, and deposition of thick sediments offshore, are suggested to induce pressure gradients that drive the flow of the ductile crust from the centre of the former rift zone towards the continental interior^27^ (Fig. 7e), possibly linking the observed accelerated Quaternary subsidence and the simultaneous onshore surface uplift^10^.

The North Sea is characterised by a lower topography (i.e. sea level), water and low-density sediments and a thinner crystalline crust under the basin (13–15 km) than the surrounding landmasses with crustal thicknesses between 32–50 km^47^. Thus, while sediment loading and onshore erosion create a pressure increment directed away from the basin, it is hard to see how the net lithostatic pressure in the lower crust should become higher beneath the basin. Even if lower crustal flow is of local significance in the North Sea^10,17^, it is unlikely on its own to explain the residual subsidence during the Quaternary relative to the surrounding highs.

Alternatively, metamorphic reactions and alteration of the lower crust were proposed as possible mechanisms for subsidence in the North German Basin and surrounding regions^12^. During the Early Permian, for instance, an increase in heat flow of 10% is proposed to have triggered lower crustal metamorphic processes within the North German Basin. The resulting transition from greenschist to amphibolite facies is expected to increase rock densities and reduce rock volumes, resulting in increased subsidence^12^. However, during the Quaternary, there is no indication for a thermal anomaly in the North Sea Basin and this process is equally considered to be unlikely.

### **I**ntraplate stresses and lithospheric buckling

**Figure 8:**
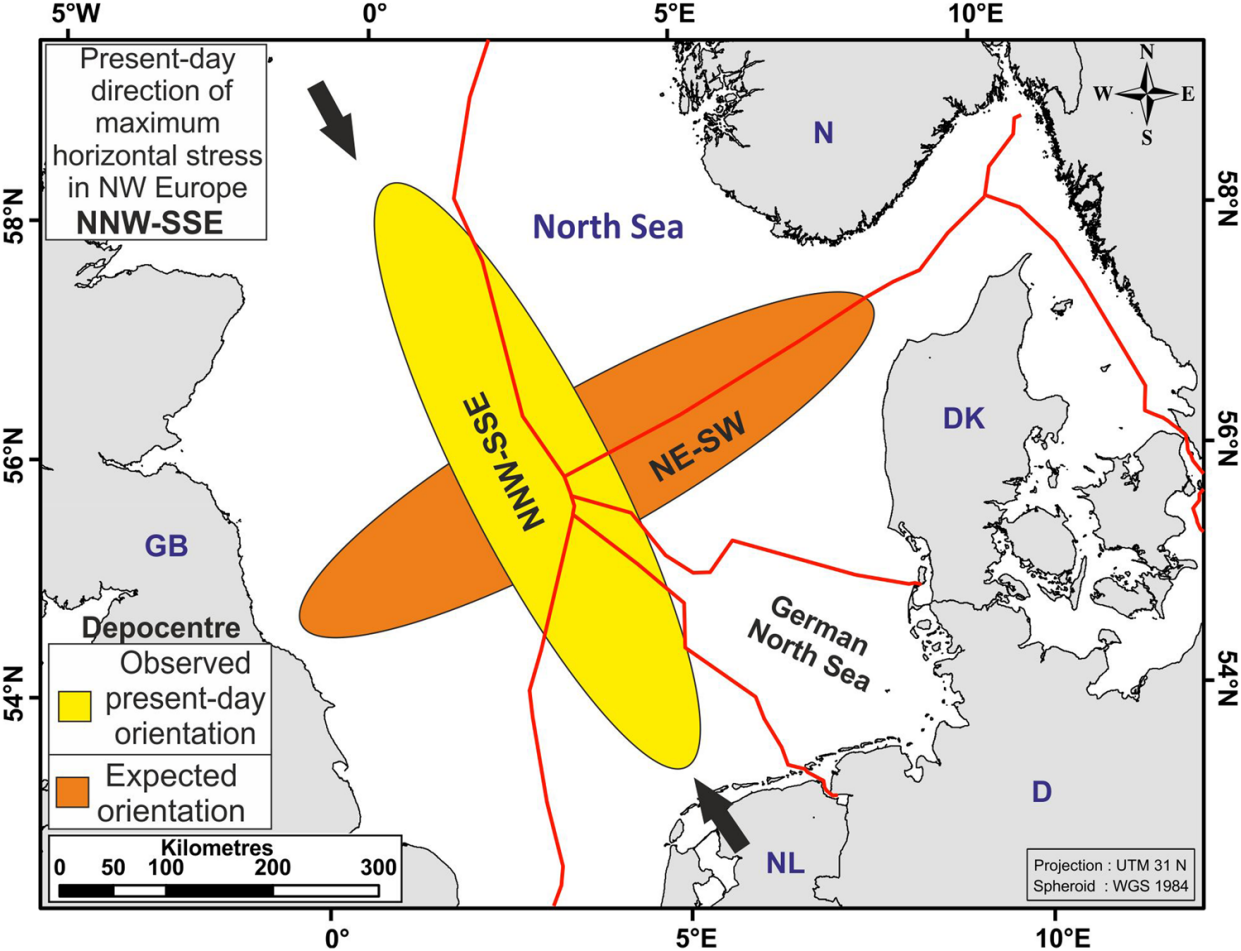
The Quaternary depocentre follows an axis along the central North Sea (yellow ellipse) with a NNW-SSE orientation approximately parallel to the present-day stress direction in NW Europe. Buckling of a mechanically isotropic lithosphere is expected to result in an approx. NE-SW oriented depocentre, perpendicular to the observed present-day trend (orange ellipse). The North Sea Central Graben as a major mechanical discontinuity is not suitably oriented for strong reactivation in the present-day stress field. Land polygons ©OpenStreetMap contributors^65^ (available under the Open Database License; see www.openstreetmap.org/copyright).

Intraplate compressional stresses have been proposed to cause ‘buckling’, or flexure of the lithosphere, resulting, in combination with sediment loading and compression-induced deep lithospheric phase reactions, in enhanced local subsidence^8,11,12^. 3D flexural modelling suggests that compressive intraplate forces, e.g. from the Atlantic ridge push or the Alpine convergent plate boundary, and subsequent lithospheric buckling can account for up to 700 m of accelerated subsidence in the North Sea^8^ (Fig. 7f). This model response was forced, however, by a prescribed area of strongly decreased effective elastic thickness (down to 3 km from a background value of 15 km) with no close relationship to the known crustal structure^8^. The orientation (NNW-SSE) of the main Quaternary depocentre (Figs 1 and 8) was also obtained via the orientation of the weak zone, whereas horizontal stress was modeled as isotropic. We consider such a process to be unlikely to contribute to the residual subsidence due to the orientation of the Quaternary depocentre. If intraplate stress-driven lithospheric buckling affected the North Sea region, then the Quaternary depocentre would be expected to have a NE-SW trend, approximately perpendicular to the observed present-day direction of maximum horizontal stress^58,59,63^ (Fig. 8). It is also surprising that such an inevitable major tectonic event had no expression in the sedimentary successions by e.g. compressional-related reactivations of faults^8,11,64^, if buckling of the lithosphere with major compressive forces took place while Quaternary sediments were deposited.

In conclusion, the complex structural evolution of the North Sea since the Caledonian mountain building phase with several subsequent extensional and compressional tectonic events alongside the former suture zones, accompanied by sediment deposition and erosional processes, sea level fluctuations – particularly as a result of glaciation and deglaciation phases during the Quaternary – make it difficult to decipher the origin and to quantify individual processes leading to the differential subsidence. However, the main outcome of this study is that the major portion (~75%) of the observed overall Quaternary subsidence is explained by load-induced subsidence and compaction, with other processes playing a minor role.

## Conclusions

Rapid Quaternary subsidence in the North Sea Basin and the origin of the substantial accommodation space created during this period are investigated. Our study is based on 2D and 3D reflection seismic surveys, calibrated by exploration wells, covering northwestern offshore Germany, and is supplemented by results from regional studies. Iceberg scour marks, which are distinct in the seismic data, form our main chronostratigraphic markers. The main results of this study are:

- Quaternary reflection surfaces reveal distinct iceberg scour marks in northwestern offshore Germany, indicating shallow water depths (<100 m) during formation. A correlation of two of the horizons with results from a high-resolution 2D-seismic survey, calibrated by well data from the Dutch offshore, reveals ages of 1.9 and 2.6 Ma.
- Using the oldest well-calibrated Quaternary horizon, a maximum local subsidence rate of up to 480 m/Ma is derived, which indicates a more than ten-fold Quaternary increase in subsidence relative to average Cenozoic rates.
- The present-day depth of this Quaternary surface in northwestern offshore Germany attains a maximum local depth of 1100 m. The Quaternary isopach map illustrates an increasing subsidence trend from the southeastern part of the study area towards the central North Sea Basin. However, this Quaternary depocentre is clearly offset from the Jurassic Central Graben in offshore Germany.
- Most of the subsidence can be explained by compaction and load-induced subsidence. Sediment loading explains up to 665 m of subsidence, and thus about half of the observed maximum Quaternary subsidence. Polygonal fault systems in the Lower Neogene and Paleogene successions are interpreted as expressions of compaction below the Quaternary sedimentary succession. A range of compaction between 150–250 m for the Lower Neogene and Palaeogene strata is calculated. Thus, loading by sediment and water in conjunction with compaction explain approximately 75% of the observed total subsidence.
- We rule out renewed extensional deformation as an explanation for the remaining 25% of the Quaternary subsidence. With the exception of isolated top diapiric faults above active salt domes, there are no significant indications for Quaternary normal faults in our extensive data. Subsidence due to salt movement is unlikely to be the cause because the Quaternary depocentre is clearly offset from the salt-dominated Mesozoic rift structures.

Intraplate stress-driven lithospheric buckling is also an unlikely cause because the main Quaternary depocentre is elongated along a NNW-SSE axis in the North Sea Basin, parallel to the major horizontal stress, and not perpendicular to it as expected. Mechanisms that cannot be excluded from contributing to the residual subsidence are post-glacial collapse of the glacier forebulge after the retreat of glaciers in the North Sea Basin, local lower crustal flow, and dynamic topography caused by sub-lithospheric mantle processes. Due to the dynamic structural evolution of the North Sea Basin, it remains unclear if there is a single origin or a combination of different processes to explain the 25% of residual subsidence.

## Methods and Stratigraphic Correlation

A suite of four commercial 3D seismic surveys covering an area of ~4000 km² in northwestern offshore Germany referred to as the Entenschnabel area, (Fig. 2) have been used to investigate the geomorphology and ice-related glacial features of the Quaternary succession. The time-migrated 3D seismic datasets have a bin size of 12.5 m and are sampled with 4 ms. Two horizons with iceberg scour marks for Quaternary subsidence analysis are used. Iceberg scour marks were interpreted on eight horizons representing the former Quaternary high-latitude sea floor of the North Sea, whose base surface is buried to a present-day depth of more than 1000 m (Figs 4 and 5).

The stratigraphic correlation is based on interpreted seismic horizons from a high-resolution 2D seismic survey (SNST-NL-87-3c, see Fig. 2 for location) from the Dutch offshore area^40^ which are tied to a detailed age model based on wells A-15-3 and B-13-3^40^ (Figs 2 and 4). 10 horizons are traced through the 3D seismic survey into northwestern offshore Germany, and two of them are presented in this study.

The time-depth conversion of interpreted seismic reflectors is based on a generalized linear relationship that reflects depth due to burial and compaction^54^. Standard backstripping methods^46^ and data from ~29 well locations are used for subsidence analysis. However, in exploration wells, the uppermost Cenozoic section is typically not studied in great detail, and the adopted lithologies are mostly generalized.

Sedimentation rates and compaction of the Cenozoic successions are calculated based on a 3D basin and petroleum system model^53^ and generalized lithologies adopted from well information.

The flexural response of the crust to loading^49,50^ is calculated using the 2D flexure calculation program Flex2D version 4.4 which is freely available on the website http://www.ux.uis.no/~nestor/work/programs.html.

### Data availability

The multichannel seismic surveys analysed during the current study are not publicly available due to limited data access for the deep subsurface of offshore Germany. 14 depth converted surfaces derived from reflection seismic datasets from the base Zechstein to Present^54^, and a 3D basin and petroleum system model^53^ used for compaction and subsidence analysis during the Quaternary, are available online and can be downloaded from the website www.GPDN.de.
